# Developmenrt of EST-SSR and genomic-SSR markers to assess genetic diversity in *Jatropha Curcas L.*

**DOI:** 10.1186/1756-0500-3-42

**Published:** 2010-02-24

**Authors:** Mingfu Wen, Haiyan Wang, Zhiqiang Xia, Meiling Zou, Cheng Lu, Wenquan Wang

**Affiliations:** 1 Institute of Tropical Biosciences & Biotechnology, Chinese Academy of Tropical Agricultural Science, 571101, Haikou, Hainan, PR China; 2 College of Agronomy, Hainan University, 571737, Danzhou, Hainan, PR China

## Abstract

**Background:**

*Jatropha curcas L. *has attracted a great deal of attention worldwide, regarding its potential as a new biodiesel crop. However, the understanding of this crop remains very limited and little genomic research has been done. We used simple sequence repeat (SSR) markers that could be transferred from *Manihot esculenta *(cassava) to analyze the genetic relationships among 45 accessions of *J. curcas *from our germplasm collection.

**Results:**

In total, 187 out of 419 expressed sequence tag (EST)-SSR and 54 out of 182 genomic (G)-SSR markers from cassava were polymorphic among the *J. curcas *accessions. The EST-SSR markers comprised 26.20% dinucleotide repeats, 57.75% trinucleotide repeats, 7.49% tetranucleotide repeats, and 8.56% pentanucleotide repeats, whereas the majority of the G-SSR markers were dinucleotide repeats (62.96%). The 187 EST-SSRs resided in genes that are involved mainly in biological and metabolic processes. Thirty-six EST-SSRs and 20 G-SSRs were chosen to analyze the genetic diversity among 45 *J. curcas *accessions. A total of 183 polymorphic alleles were detected. On the basis of the distribution of these polymorphic alleles, the 45 accessions were classified into six groups, in which the genotype showed a correlation with geographic origin. The estimated mean genetic diversity index was 0.5572, which suggests that our *J. curcas *germplasm collection has a high level of genetic diversity. This should facilitate subsequent studies on genetic mapping and molecular breeding.

**Conclusion:**

We identified 241 novel EST-SSR and G-SSR markers in *J. curcas*, which should be useful for genetic mapping and quantitative trait loci analysis of important agronomic traits. By using these markers, we found that the intergroup gene diversity of *J. curcas *was greater than the intragroup diversity, and that the domestication of the species probably occurred partly in America and partly in Hainan, China.

## Background

*Jatropha curcas L. *is a perennial, monoecious shrub that belongs to the *Euphorbiaceae *family. The species is native to America but is distributed widely in the tropics [1,2]. Wild or semicultivated types of *J. curcas *can grow well under all unfavorable climatic and soil conditions [3]. The seeds of *J. curcas *contain 40-45% oil [4-6] with a high percentage of monounsaturated oleic and polyunsaturated linoleic acid. The seed oils can be classified as semi-drying [7]. In recent years, the economic importance of *J. curcas *for the production of biodiesel fuel has been increasingly recognized [8].

To use *J. curcas *for producing biofuel, it is crucial to develop varieties with a high seed yield and a high oil content that are adapted well to varied conditions [9]. In recent years, *J. curcas *germplasm has been collected and analyzed in Brazil, India, Indonesia, and China [10,11]. *J. curcas *has a heterozygous genome, so conventional breeding programs for its improvement might not be effective. Hence, it is likely that genomics-based breeding strategies need to be used. However, the genetic map of *J. curcas *is not well-developed and very limited information is available with respect to molecular markers. The traditional methods of developing simple sequence repeat (SSR) markers are usually time-consuming and labor-intensive. However, an alternative strategy has been developed that uses comparative genomics to identify SSR markers. This strategy has been used successfully in barley [12] and *Brassica *[13].

In the study reported herein, we selected 419 expressed sequence tag (EST)-SSR and 182 genomic (G)-SSR primer pairs that had been developed for *Manihot esculenta *(cassava), which also belongs to the *Euphorbiaceae *family, and investigated whether they could be transferred to *J. curcas*. Firstly, these primer pairs were tested using five accessions of *J. curcas*. The primer pairs that produced specific amplicons were then tested further. Finally, we used the transferable markers to analyze the genetic diversity of our collection of *J. curcas *accessions.

## Results

### Identification of EST-SSRs and G-SSRs for use in *J. curcas*

**Figure 1:**
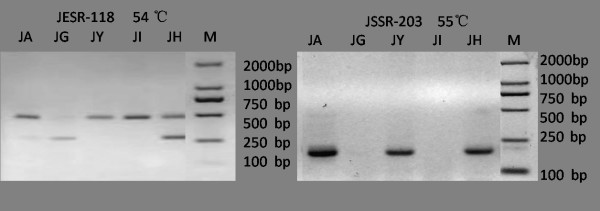
**PCR amplification of the loci JESR-118 (CESR0806) and JSSR-203 (SSRY100) in five accessions of *Jatropha curcas***.

Five accessions of *J. curcas *were used to test the transferability of 419 EST-SSR and 182 G-SSR primer pairs between cassava and *J. curcus*. Among these primers, 234 (55.85%) EST-SSR and 68 (37.36%) G-SSR primer pairs produced amplicons, but only 187 (44.63%) EST-SSRs and 54 (29.67%) G-SSRs were polymorphic among the five accessions (Fig.1). For each of the 241 EST-SSR and G-SSR markers in *J. curcas*, the name, sequence of the forward and reverse primers, the repeat type, annealing temperature, and expected size of the PCR products are listed in additional file 1.

### Characteristics of the EST-SSR and G-SSR markers and relevant genes in *J. curcas*

Microsatellites or SSRs are regions in the genome of tandemly repeated DNA segments, with each segment comprising up to six bases. Analysis of the nucleotide sequences of the EST- and G-SSRs showed that, in *J. curcas*, the EST-SSRs corresponded to 57.75% trinucleotide repeats, 26.20% dinucleotide repeats, 8.56% pentanucleotide repeats, and 7.49% tetranucleotide repeats. In contrast, the G-SSRs were composed mainly of dinucleotide repeats (62.96%) (Table 1).

**Table 1:** Structural characteristics of the ES T-SSR and G-SSR markers in *J. curcas*

Repeat type	EST-SSR			G-SSR		
	
	No.	Polymorphic		No.	Polymorphic	
				
		Number and percentage			Number and percentage	
Di	57	49	26.20	43	34	62.96
Tri	139	108	57.75	4	2	3.70
Tetra	20	16	8.56	-	-	-
Penta	18	14	7.49	-	-	-
Total	234	187	100	68	54	100

**Figure 2:**
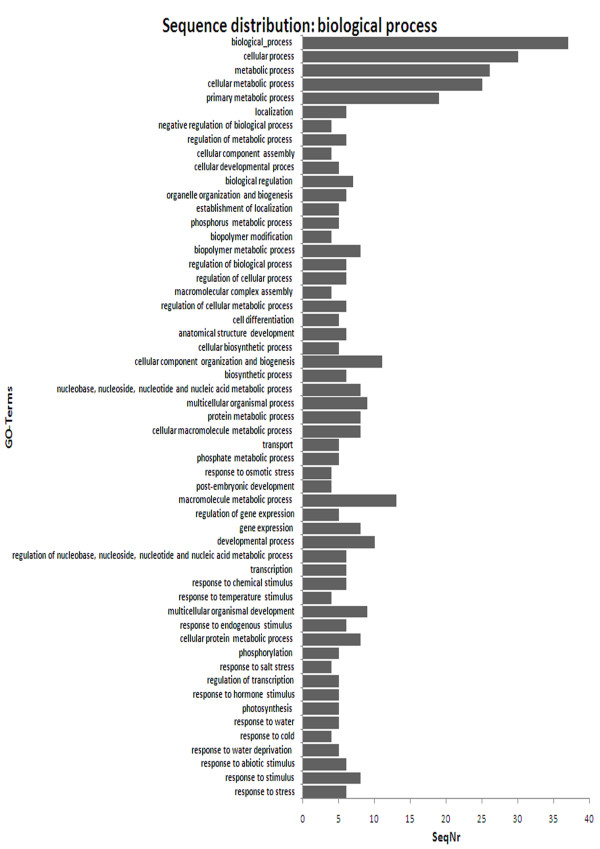
**Functional classification of the ESTs amplified by the 187 EST-SSR primer pairs in *J. curcas *(GO-Terms: Gene Ontology Terms)**.

A BLAST search of the GenBank database was performed using the sequences of the 187 EST-SSRs. The functions of the ESTs were then clustered according to the general functional categories in the KOG (Eukaryotic Orthologous Groups of Proteins) database. A general functional classification of the EST-SSRs is shown in Fig. 2. Out of the 187 unique ESTs, 184 were annotated and they were classified into more than 55 categories. Many ESTs were found in several categories. The five main categories, which contained 137 of the ESTs, were biological processes, cellular processes, metabolic processes, cellular metabolic processes, and primary metabolic processes.

### Assessment of genetic diversity in *J. curcas*

Thirty-six EST-SSRs and 20 G-SSRs were used to estimate the genetic diversity of 45 accessions of *J. curcas*. A total of 216 alleles were identified, and 183 (84.72%) of them were polymorphic. The sizes of the amplicons for the EST-SSRs and G-SSRs ranged from 120 to 600 bp. The 36 EST-SSRs generated 152 alleles, of which 128 (84%) were polymorphic, whereas the 20 G-SSRs produced 64 alleles, of which 55 (86%) were polymorphic. The number of alleles for each G-SSR ranged from one to six with a mean of 3.20; in contrast, the number of alleles for each EST-SSR ranged from one to nine with a mean of 4.22. Therefore, more alleles were obtained with the EST-SSRs than with the G-SSRs.

**Figure 3:**
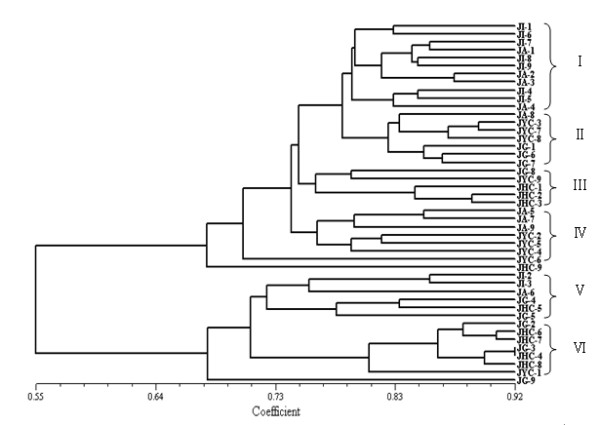
**An UPGMA tree of the 45 *Jatropha *accessions**.

Genotyping data that were obtained for all 183 polymorphic alleles were used to estimate pairwise similarity comparisons among these accessions. The similarity coefficient values of the phenogram ranged from 0.55 to 0.92 with a mean of 0.76 (Fig. 3). The 45 accessions clustered into six groups: group I, which comprised seven accessions from Indonesia and four from South America; group II, which comprised three accessions from Grenada, one from South America, and three from Yunnan, China; Group III, in which all four accessions were from Hainan, China; Group IV, which comprised four accessions from Yunnan, China plus three American clones; Group V, which consisted of two accessions from Indonesia, two from Grenada, one from America, and one from Hainan, China; and Group VI, which comprised four clones from Hainan, China, three from Grenada, and one from Yunnan, China. The results suggested that, in general, the genotypes of the *J. curcas *accessions are correlated with their geographic origins, although some crosses had occurred.

The parameters of genetic diversity between and within the five geographic groups (Table 2) were estimated using all 183 EST-SSR and G-SSR polymorphic alleles. The genetic diversity index ranged from 0.4099 to 0.5022 with an average of 0.5572, which suggested that the collection had a broad genetic background, although the level of genetic diversity was higher among the accessions from America and Yunnan, China. The total gene diversity (Ht = 0.3819 ± 0.0197) was higher than the gene diversity within groups (Hs = 0.3108 ± 0.0158), which indicated that the genetic diversity between groups is greater than that within groups. The coefficient of gene differentiation (Gst) was 0.1861, which indicated that the level of genetic differentiation among the five groups was relatively high. The gene flow index (Nm) was 2.18, which indicated that high levels of gene flow occurred during the domestication of *J. curas*.

**Table 2:** Parameters of intra- and intergroup genetic diversity among the five populations of *J. curcas*

Population	n	#loc_P	PLP (%)	na	ne	H	I
Indonesia	9	118	54.63	1.7541	1.5769	0.3140	0.4526
Grenada	9	100	46.30	1.6995	1.5046	0.2818	0.4099
South America	9	147	68.06	1.8907	1.5763	0.3326	0.4920
Yunnan, China	9	108	50.00	1.8361	1.6274	0.3473	0.5022
Hainan, China	9	148	68.52	1.8142	1.4575	0.2785	0.4221
Total	45	183	84.72	2.0000	1.6869	0.3819	0.5572
St. Dev	-	-	-	± 0.0000	± 0.3107	± 0.1403	± 0.1741

				**Ht**	**Hs**	**Gst**	**Nm**

Mean				0.3819	0.3108	0.1861	2.1868
St. Dev				± 0.0197	± 0.0158		

n = number of genotypes per sample;

#loc P = number of polymorphic loci;

PLP = percentage of polymorphic loci;

na = observed number of alleles;

ne = effective number of alleles [Kimura and Crow (1964)];

h = Nei's (1973) gene diversity;

I = average genetic diversity index;

Ht = total gene diversity;

Hs = intrapopulation gene diversity;

Gst = coefficient of gene differentiation;

Nm = estimate of gene flow from Gst; Nm = 0.5 (1-Gst)/Gst

**Figure 4:**
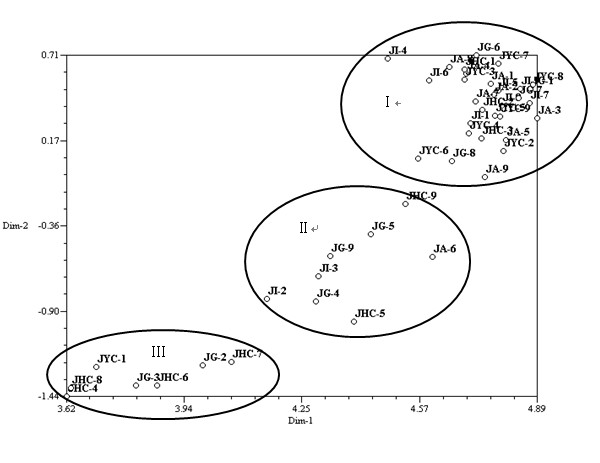
**Two-dimensional PCA plot of the 45 *J. curcas *accessions**.

The genetic similarity matrix that was obtained from Jaccard's similarity coefficient was subjected to principal coordinates analysis (PCA). The results indicated that all 45 accessions could be divided into three categories (Fig. 4). Category I contained the most accessions and they came from all five areas. Category II included eight accessions in total: three from Grenada, two from Indonesia, two from Hainan, and one from America. Category III contained four accessions from Hainan, two from Grenada, and one from Yunnan. Category I included many accessions from different areas, and therefore demonstrated the weak geographic differentiation in the population; in other words, the observed genetic diversity was due mainly to original evolution. The category II and category III clusters revealed that the phylogenetic characteristics of *J. curcas *from South America, where *J. curcas *originated, and from Hainan in China, where the species was domesticated less than 300 years ago, were very similar.

## Discussion

### Development of molecular markers in *J. curcas*

It was crucial to develop a sufficient number of species-specific markers to enable the fingerprinting and genetic mapping of *J. curcas*; in rice and soybean, for example, more than 10,000 SSR and SNP markers are available [14,15]. Random amplified polymorphic DNA (RAPD), inter-simple sequence repeat (ISSR) and amplified fragment length polymorphism (AFLP) markers have been used to evaluate genetic diversity and for fingerprinting in accessions of *J. curcas *and related species [11,16-18]. Up to now, only 12 SSR markers had been developed for *Jatropha *(they are polymorphic in six species of *Jatropha*) [19], and no genetic map of *Jatropha *has been reported. The transferable SSRs identified in the present study should enable the genetic diversity, elite clones, and evolution of *J. curcas *to be assessed, and a linkage map to be constructed.

### Transferability of EST-SSRs and G-SSRs from cassava

The development of EST-SSR markers was particularly attractive because they represent coding regions of the genome. It has been reported that EST-derived SSRs and G-SSRs show a considerable degree of transferability to related species [20-24]. In the present study, we observed a high level of transferability from cassava to *J. curcas*; the level of transferability was higher for EST-SSRs (44.63%) than for G-SSRs (29.67%). This result was consistent with that reported for wheat and related species [25]. In several studies, the level of transferability of G-SSRs was found to be only 20-30% [26-29]. The higher levels of transferability of EST-SSRs than of G-SSRs reflect the conserved nature of coding sequences as compared with non-coding genomic DNA, and the fact that the mutation frequency of EST sequences is lower than that of genomic DNA sequences. These results demonstrate the potential value of EST-SSR markers for the development of genetic maps, assessment of genetic diversity, and marker-assisted selection (MAS) breeding in *J. curcas*, all of which would benefit comparative mapping and analysis of the comparative functions of genes among the economic species in the *Euphorbiaceae *family. Our finding that the majority of transferable EST-SSRs were trinucleotide repeats agreed with previous studies [21,30-32], and can be explained by the suppression of non-trimeric SSRs in coding regions due to the risk of frameshift mutations, which might occur with non-trimeric microsatellites [33-36].

### Collection of germplasm and evaluation of genetic diversity

Several projects to collect germplasm have been carried out in Brazil [37], India [38], and China [39], but systematic work on the collection of germplasm and its evaluation is still in its infancy. The low genetic variability found among accessions of *J. curcas *from Africa and Asia [11,16,17] has demonstrated the need for new sources of genetic variation in *J. curcas *that could be used in breeding programs. Such sources of genetic variation have been identified in Latin America, especially in Guatemala [40]. In the present study, high levels of genetic diversity were revealed in the 45 *Jatropha *accessions analyzed with the 56 EST-SSR and G-SSR primer pairs that were utilized. The accessions from South America, Yunnan (China), and Indonesia showed higher levels of genetic variation than the other two geographic regions (Grenada and Hainan, China). In particular, the collections from Yunnan (China) could be used to enrich the genetic background of *J. curcas *for breeding. Gst and Nm showed that some differentiation occurred in each geographic group.

## Conclusion

We developed a set of 241 SSR primer pairs for use in *J. curcas*. Of these markers, 187 EST-SSRs should be useful for genetic mapping and quantitative trait loci analysis of important agronomic traits. Fifty-six EST-SSRs and G-SSRs were used successfully to analyze genetic diversity in 45 accessions of *J. curcas*. The accessions analyzed showed a broad genetic background with an average genetic diversity index of 0.5572. The intergroup genetic diversity was larger than the intragroup diversity, and domestication had taken place in both America and Hainan, China.

## Methods

### Plant material

Five *J. curcas *accessions, which corresponded to JI-1(Indonesia), JA-1(America), JG-1 (Grenada), JYC-1(Yunnan, China), and JHC-1(Hainan, China), were used to test the suitability of the cassava SSR markers by PCR. To analyze the genetic diversity of *J. curcas*, 45 accessions were chosen, nine from each of the five above-mentioned regions (Table 3). All the materials were obtained from the germplasm collection of the Institute of Tropical Biosciences & Biotechnology, Chinese Academy of Tropical Agricultural Sciences, Haikou, China. This collection contains 154 *J. curcas *accessions, which cover most of the Chinese collections as well as accessions from eight other countries: Brazil, Columbia, Grenada, Thailand, Indonesia, Laos, Myanmar, and Nigeria. Total genomic DNA was isolated from young leaves by the modified CTAB method as described by Doyle and Doyle [41]. The genomic DNA was diluted to a concentration of 25 ng/μl for PCR amplification.

**Table 3:** The *J. curcas *accessions and their origins

Code	District of collection	Code	District of collection	Code	District of collection
JI-1	Indonesia	JA-7	South America	JYC-4	Yunnan, China
JI-2	Indonesia	JA-8	South America	JYC-5	Yunnan, China
JI-3	Indonesia	JA-9	South America	JYC-6	Yunnan, China
JI-4	Indonesia	JG-1	Grenada	JYC-7	Yunnan, China
JI-5	Indonesia	JG-2	Grenada	JYC-8	Yunnan, China
JI-6	Indonesia	JG-3	Grenada	JYC-9	Yunnan, China
JI-7	Indonesia	JG-4	Grenada	JHC-1	Hainan, China
JI-8	Indonesia	JG-5	Grenada	JHC-2	Hainan, China
JI-9	Indonesia	JG-6	Grenada	JHC-3	Hainan, China
JA-1	South America	JG-7	Grenada	JHC-4	Hainan, China
JA-2	South America	JG-8	Grenada	JHC-5	Hainan, China
JA-3	South America	JG-9	Grenada	JHC-6	Hainan, China
JA-4	South America	JYC-1	Yunnan, China	JHC-7	Hainan, China
JA-5	South America	JYC-2	Yunnan, China	JHC-8	Hainan, China
JA-6	South America	JYC-3	Yunnan, China	JHC-9	Hainan, China

### Design of EST-SSR and G-SSR primers and validation in *J. curcas*

A total of 419 EST-SSR primers that had been developed for cassava in our laboratory [42] and 182 G-SSR primers [43] were synthesized and used. Amplification by PCR was performed in a 20-μl reaction mixture that contained 50 ng template DNA, 1× PCR buffer (20 mM Tris pH 9.0, 100 mM KCl, 3.0 mM MgCl_2_), 400 μM of each of the four dNTPs, 0.4 μM of each of the forward and reverse primers, and one unit of Taq DNA polymerase. The following PCR conditions were used: 94°C for 1 min, followed by 35 cycles of 94°C for 1 min, 45-57°C for 1 min, 72°C for 1 min, and 10 min at 72°C for the final extension. PCR products were separated on a 2% agarose gel, and visualized using SYBR Green http://www.Genecopoeia.com.

### Putative functional annotation of EST-SSRs

To assess the putative function of the EST-SSRs developed here, a BLASTX search of the GenBank nonredundant database http://www.ncbi.nlm.nih.gov/BLAST was performed using the 187 ESTs that contained polymorphic microsatellites. The threshold for a significant BLAST hit was set at a bit score greater than 80 bits. Functional categories were assigned on the basis of the BLAST searches using a specially formatted database of the Eukaryotic Orthologous Groups of proteins (KOG).

### Analysis of genetic diversity

The polymorphic alleles obtained with each primer pair were scored for their presence (1) or absence (0). From the data matrix, a dendrogram was constructed using the unweighted pair group method using arithmetic averages (UPGMA), the similarity coefficient, and the software NTSYS-pc2.1 [44]. The binary data were also subjected to PCA to investigate the structure of our collection. The genetic diversity parameters of each geographic group, which included the percentage of polymorphic loci (PLP), observed number of alleles per locus (na), effective number of alleles per locus (ne), Nei's gene diversity (h), Shannon's information index (I), Gst, and Nm, were calculated by POPGENE 32 [45].

## Competing interests

The authors declare that they have no competing interests.

## Authors' contributions

MW and HW contributed to the design of the study, performed the polymorphic amplification of the EST-SSR primers in *J. curcas*, and prepared the manuscript. ZX contributed to the analysis of the data. MZ identified the EST-SSR primers in cassava. CL participated in the collection of the germplasm. WW was responsible for the research and the manuscript. All authors read and approved the final manuscript.

## Supplementary Material

Additional file 1 **The EST-SSR and G-SSR markers in *Jatropha curcas***. the name, sequence of the forward and reverse primers, the repeat type, annealing temperature, and expected size of the PCR products are listed in Additional file 1. Click here for file
