# Supplementary material for: Developmenrt of EST-SSR and genomic-SSR markers to assess genetic diversity in Jatropha Curcas L
Source: BMC Res Notes. 2010 Feb 24;3:42. doi: 10.1186/1756-0500-3-42 (PMC2844395; doi:10.1186/1756-0500-3-42)
Supplement: Additional file 1 — The EST-SSR and G-SSR markers in Jatropha curcas. the name, sequence of the forward and reverse primers, the repeat type, annealing temperature, and expected size of the PCR products are listed in Additional file 1. [file 1756-0500-3-42-S1.DOC]

**Additional files**

**Additional file 1**

**The EST-SSR and G-SSR markers in *Jatropha curcas*. The name, sequence of the forward and reverse primers, the repeat type, annealing temperature, and expected size of the PCR products are listed in Additional file 1.**

| Nomination | Original Name | Sequence ID | Repeat  Type | Forward primer | Reverse primer | Annealing Tm  (°C) | Expected size  (bp) |
| --- | --- | --- | --- | --- | --- | --- | --- |
| JESR-001 | CESR-0163 | DV457345 | tri－ | AACCACAGGAGTTGGTAATG | GAAAGAAGCAACAGAAATGG | 50 | 307 |
| JESR-002 | CESR-0235 | DV453320 | penta－ | TGTCACAGTACATGCCACTT | ACCTTCGATTCATTTGTGTC | 50 | 287 |
| JESR-003 | CESR-0269 | DV454304 | tri－ | CCATCCTCAAACAGAGAGTT | TGCATCCGATTATTTCTCTT | 50 | 345 |
| JESR-004 | CESR-0285 | CK651453 | tri－ | CAGAGCTTTGCCTAACCAT | TCCATCACCAGATCCTAAAC | 50 | 228 |
| JESR-005 | CESR-0290 | DV448365 | tri－ | TCAATCAATTCCAACAACAA | CTAAATTCTCAACCAGGTGC | 50 | 192 |
| JESR-006 | CESR-0333 | DN238601 | tri－ | ACATCTACAATGGCGATTTC | TAATGAATCTGTAGGACCCG | 50 | 166 |
| JESR-007 | CESR-0361 | CK644812 | tri－ | AGGCAGATATGCTGATTAAGTT | AGGCAGATATGCTGATTAAGTT | 50 | 364 |
| JESR-008 | CESR-0460 | DV448357 | tetra－ | GAAATTTGGATCAGCATCAT | AGCATTTGGTGAGAAGAGAA | 50 | 185 |
| JESR-009 | CESR-0567 | DV447558 | tri－ | TGATTCCTCGACTTTGCTAT | GAACCAACAACAAAGCTCAT | 50 | 238 |
| JESR-010 | CESR-0703 | DR087955 | di－ | ATCAAAGTAATGGATGTCGG | TATCATCCACAGCAACCATA | 50 | 184 |
| JESR-011 | CESR-0708 | DN238894 | di－ | ATCCAAGAAGGCAGTAGTGA | CTCATGCATGGAGATACAAA | 50 | 228 |
| JESR-012 | CESR-0718 | CK644018 | tri－ | ATGATGTTAAGGACGAGGTG | ATCGGCTTCTTCTTCTTCTT | 50 | 193 |
| JESR-013 | CESR-0719 | CK644018 | tri－ | ATGATGTTAAGGACGAGGTG | GCAAGTTCTGGATTGTTCTC | 50 | 391 |
| JESR-014 | CESR-0749 | BM260234 | penta－ | CAGCAGAGTTCTGATTAGCC | TTACAGTTTGGCTTGGATCT | 50 | 230 |
| JESR-015 | CESR-0756 | DN239199 | di－ | CAGGTTCGTCTTCTTCAACT | ATATATGATCCCGACAACAA | 50 | 302 |
| JESR-016 | CESR-0804 | DV442410 | tri－ | CGTATTACCACCAACATCCT | CCATCAATCCACTTATCGTT | 50 | 205 |
| JESR-017 | CESR-0208 | DV443162 | tri－ | GTGATCTCCAGCTCAAAGAG | TCTCCTCCTTATCATTCACG | 52 | 219 |
| JESR-018 | CESR-0225 | DV456543 | di－ | TGGATGCAATCTTAGACACA | GATGGTTACAGCTCACCAAT | 52 | 399 |
| JESR-019 | CESR-0246 | CK645960 | di－ | TGGTACTGAATCTCCTGGAC | ACGATTGTCCATTCTTTGTG | 52 | 226 |
| JESR-020 | CESR-0249 | DV447605 | tetra－ | CAGTTGTTGGGATTTGAGAT | AAGGTTTACCACGTCAACAC | 52 | 199 |
| JESR-021 | CESR-0302 | CK649631 | tri－ | AAACCCACAAGCAGAGGA | ATCCTTCTCATTCTCAAGCA | 52 | 255 |
| JESR-022 | CESR-0303 | DV456980 | tri－ | AAAGCCTAGTGCATTTGAAG | TCATGCTCTTTCTCCATCTT | 52 | 347 |
| JESR-023 | CESR-0306 | CK901302 | penta－ | AAATTGGTTACTGGCCTGA | GCTGATAAATCTTGCTTTGC | 52 | 153 |
| JESR-024 | CESR-0310 | DV450642 | di－ | AACCATTTGATTTGATGGAG | CCACTTGGTCATGAGAGAAT | 52 | 290 |
| JESR-025 | CESR-0312 | CK644133 | tri－ | AAGAAACAAACAATTGTGCC | TAGAAATCCTTGCTGGGTTA | 52 | 377 |
| JESR-026 | CESR-0313 | CK644593 | tri－ | AAGAAATACAAGCAGGGTCA | TGCATCAAATGGTACAGAGA | 52 | 282 |
| JESR-027 | CESR-0330 | DV448635 | tri－ | ACATCAATAAACCAGATGCC | ACCAACTGTTCCAAATTGAC | 52 | 266 |
| JESR-028 | CESR-0344 | DV446698 | penta－ | ACTTCCTTCAGATCATGCAC | CTGGGTAATCTTGTTCCAAA | 52 | 292 |
| JESR-029 | CESR-0356 | DV453050 | penta－ | AGGACCATATTGCTGATGAC | TAATGACGATGACAACAAGC | 52 | 233 |
| JESR-030 | CESR-0370 | CK644798 | di－ | ATCAATGAACCTGTTGAAGG | TCCAGACTCCAAACTTAATCA | 52 | 359 |
| JESR-031 | CESR-0382 | DV458464 | tri－ | ATGACCTACAAGCAAGCACT | CCAATGACACCTCAGAATTT | 52 | 343 |
| JESR-032 | CESR-0386 | DV451083 | tri－ | ATTACTGTGAATGAAGCCCA | ACAGAGCACCCTAACGATAA | 52 | 325 |
| JESR-033 | CESR-0393 | DV451766 | tri－ | CAACAGCAAACTTCCTTCTT | TTTGCACTGTCACCAAGAT | 52 | 320 |
| JESR-034 | CESR-0398 | DV445145 | tri－ | CAATCTTATTGCAGTGCTGA | CAAACAGCTAGAATCCCTTG | 52 | 366 |
| JESR-035 | CESR-0399 | CK645347 | di－ | CAATGCATGGATCATAAGTG | CTCAAGTCAAATCTGGGAAC | 52 | 330 |
| JESR-036 | CESR-0417 | DV447323 | tri－ | CATGTTACTCAGCAGAAGCA | TTGTTTGAAACGTTGCTTTA | 52 | 235 |
| JESR-037 | CESR-0448 | DV448664 | tri－ | CTGACCAGAAAGCAAAGAAC | AGAAGTTGCATCTCTTGCAT | 52 | 209 |
| JESR-038 | CESR-0637 | CK648030 | tri－ | AATGAGAATCCTTGCTGAAT | TAAGCGACAGGAACAAGAAT | 52 | 138 |
| JESR-039 | CESR-0638 | DV448343 | tri－ | AATGGTCAGAATCTTGATGG | ACCACCACCACCACCATA | 52 | 189 |
| JESR-040 | CESR-0702 | DV457522 | di－ | ATATTTATGCTCGCTTCCTG | GTACCAGACACATGAATCCC | 52 | 199 |
| JESR-041 | CESR-0705 | DR084495 | di－ | ATCAAAGTAATGGATGTCGG | TAGCAAATGGACCTCTCTCT | 52 | 100 |
| JESR-042 | CESR-0730 | DV442874 | di－ | ATTGTTAAGAACAGCACCGT | TGCATGCCACTGATTTATTA | 52 | 168 |
| JESR-043 | CESR-0768 | DV446454 | tri－ | CCAAATCACACCATTTCTTT | TGTTTGATATAGGGTTTGGC | 52 | 279 |
| JESR-044 | CESR-0836 | CK646221 | tri－ | CTTCCGCCTTCTCTTCCC | CATGGTTATCAGCCCATAGT | 52 | 366 |
| JESR-045 | CESR-0844 | DV448767 | tri－ | GAAATAGAGACAGAAGGCGA | AAATGAACAGGGAATTGTTG | 52 | 369 |
| JESR-046 | CESR-0889 | CK650219 | tetra－ | GCTCTTGATTGATTCCATTT | TGATGATGATGATGATGAGG | 52 | 391 |
| JESR-047 | CESR-0936 | DV445668 | penta－ | GTTGATACTGGAAGTGAGCC | TGTGTTCAAAGGTGATGAGA | 52 | 398 |
| JESR-048 | CESR-0987 | DR085299 | tri－ | TCTTCCGCCTTCTCTTCC | TGGATATCTAGTTCCATCCG | 52 | 256 |
| JESR-049 | CESR-1022 | DV449573 | tri－ | TTCAAGTATCCAACCATTCC | AATACCAGCCAACAAAGAAA | 52 | 308 |
| JESR-050 | CESR-1027 | DV450779 | tri－ | TTCCAATTCACCAGCCAT | ACCATGAGGATCAAGAACTG | 52 | 309 |
| JESR-051 | CESR-1044 | DV445169 | tri－ | TTGTCGAAGCTAAGGATTTC | CCATTCTTTCTTCCTTTGTG | 52 | 391 |
| JESR-052 | CESR-1049 | CK644602 | penta－ | TTTATCTTCGTGAGGTTCGT | AAACAGCTAACCCTAAATAATTC | 52 | 283 |
| JESR-053 | CESR-1050 | CK642867 | di－ | TTTCCACACATCAGCGGC | ATAAACCTTCAAACGAGCAA | 52 | 398 |
| JESR-054 | CESR-1055 | DV456145 | di－ | TTTGAGAGGTGGCAATAACT | GTCACAACCGGCAATTAG | 52 | 345 |
| JESR-055 | CESR-0061 | DV454672 | tri－ | CTTCACATCCCAAATCCA | GATCTAGCAAATGATCGGAC | 53 | 202 |
| JESR-056 | CESR-0098 | DV441753 | tri－ | CCAAATCAATGAAGAAATCC | GAGCTAGCTTCCAACAAGAA | 53 | 353 |
| JESR-057 | CESR-0212 | DV454825 | tri－ | CGTTAATCGTTTGCTTCTTT | CCTCCTGAACTTGCTTCTTA | 53 | 196 |
| JESR-058 | CESR-0231 | CK644059 | tetra－ | GCTTTAGCAAACCAAGATTC | AATCATCGTTATCGTTGGAC | 53 | 309 |
| JESR-059 | CESR-0252 | DV441864 | di－ | CATTCGTGTTGCTTAAATGA | TTCTCGGTCTTCTACCACAT | 53 | 190 |
| JESR-060 | CESR-0254 | CK647302 | penta－ | ACCTTTCCTATTGCCTTACC | CATGAAACTTCACAAGCCTA | 53 | 182 |
| JESR-061 | CESR-0264 | DV442290 | tri－ | TCAATCAATTCCAACAACAA | AGGTCCCTCCTCTGATTTAG | 53 | 385 |
| JESR-062 | CESR-0293 | DV441846 | tri－ | CAAAGAAGCCATTTCTGTTC | TATCATCACAAAGGTGCAAA | 53 | 169 |
| JESR-063 | CESR-0427 | DR086796 | di－ | CCATTTGATTTGATGGAGTT | ACAAGCCATTCTCCTACTCA | 53 | 176 |
| JESR-064 | CESR-0445 | CK646956 | penta－ | CTCTCATGGCTTCCCATT | CTTGCCGAAAGAACTAGAAA | 53 | 119 |
| JESR-065 | CESR-0453 | DV455052 | penta－ | CTTCTGAAAGAGAATGGCAC | AGAATATTCAAGCACACCAAA | 53 | 369 |
| JESR-066 | CESR-0456 | CK651283 | di－ | CTTGCAAGCCCTTCTCTAA | CTTGATTGTGGTTTCCTGAT | 53 | 148 |
| JESR-067 | CESR-0494 | DV443968 | tri－ | GCCAAAGCTTCTCTTAACAA | TAATCACCATACCCACCTTC | 53 | 287 |
| JESR-068 | CESR-0498 | CK644911 | tri－ | GCTCGTTCATTCATCTCACT | TTAACTGTGAATGCCCTTCT | 53 | 300 |
| JESR-069 | CESR-0510 | DR086106 | tri－ | GGATTTGAATGAGGTCAGAA | AAACTCAACCAAACCACAAC | 53 | 202 |
| JESR-070 | CESR-0717 | DV443630 | tri－ | ATGATCTGGTGGCTGAATAC | TGACTTTGACCATTCAACAA | 53 | 170 |
| JESR-071 | CESR-0733 | DV444088 | di－ | CAAACAGATCAAGTACCCAGA | AAATTGATGGTGGTGATGAT | 53 | 258 |
| JESR-072 | CESR-0820 | DV456878 | di－ | CTCGAGCACATGTTTAATGA | TGAGATCAATTCAACCACAA | 53 | 244 |
| JESR-073 | CESR-0870 | DV441659 | tri－ | GATGTGGCTCAAAGAAGTTT | TTCAGAAATGCTGGATCTTT | 53 | 178 |
| JESR-074 | CESR-0877 | DV453515 | di－ | GCAAGACCAGAAGCTGTATT | TCATTTCATTTCATTGCATC | 53 | 370 |
| JESR-075 | CESR-0934 | DR087664 | tri－ | GTTATTATCCTCGCCACTTG | TTTGTATTTGTTCACACGGA | 53 | 275 |
| JESR-076 | CESR-0938 | DV451549 | penta－ | GTTTCATCGATTGCTTCTTC | CTTGCAGAGAATCAACACAA | 53 | 332 |
| JESR-077 | CESR-0949 | DV447263 | di－ | TAGAGCTAATGAAACGAGCC | TTTCTCACCAAATTTCCTTC | 53 | 384 |
| JESR-078 | CESR-1006 | DR086439 | tri－ | TGGAGGATGTTCAAGTTACC | ATGGGAATGCATAATCAAAG | 53 | 365 |
| JESR-079 | CESR-1034 | CK647699 | tri－ | TTCTCTTCCTCTTGCTCATC | AGCATGGTTAACGATTTCAG | 53 | 227 |
| JESR-080 | CESR-1041 | DV457138 | tri－ | TTGCTGAAGCCCTTTCTAT | CAGTGTTGAGATCATAGCGA | 53 | 276 |
| JESR-081 | CESR-1042 | DR086637 | di－ | TTGGATTCCCTATGAACAAC | TTTGTCTGTCGAATCCTCTC | 53 | 301 |
| JESR-082 | CESR-0027 | CK644467 | tri－ | ACCAGAAGAAATCCATCTCA | GACGATGTAGATTCCCTTGA | 54 | 270 |
| JESR-083 | CESR-0043 | CK651882 | di－ | ACAGCCTCGTCATTTCACT | TAATGAATGGTTCGTAGCCT | 54 | 168 |
| JESR-084 | CESR-0069 | CK650278 | tri－ | GAAGAACATAGATGCCAAGC | TCTCCTCCTTATCATTCACG | 54 | 313 |
| JESR-085 | CESR-0072 | DV456160 | tri－ | GGAATGCTGGATATTATGGA | TCATCAGTTGAAAGCACAAG | 54 | 198 |
| JESR-086 | CESR-0078 | DV457968 | di－ | TCCCTCTCCTTCAGATTAAA | ATGATAGCCAAACAGCAACT | 54 | 333 |
| JESR-087 | CESR-0102 | DV448667 | tri－ | GATTGATGCCACTCAGGTAT | TAGGAGACAAGACATTGTGC | 54 | 219 |
| JESR-088 | CESR-0104 | CK644302 | di－ | CCCTCCCTTTGGTTTCTG | GGAGGAAAGGAGAGGAAATA | 54 | 192 |
| JESR-089 | CESR-0115 | DR084616 | tri－ | AACAACTGGTTGTGGAGTTC | TTGATGCTGTGGATATGAGA | 54 | 394 |
| JESR-090 | CESR-0207 | DV455029 | di－ | TGACATTTGTCAGTCTTGGA | TCACCATACCACACAATCAC | 54 | 227 |
| JESR-091 | CESR-0205 | CK649061 | tri－ | GAAGAACATAGATGCCAAGC | CGGATGGGTTAGAAATAGAA | 54 | 250 |
| JESR-092 | CESR-0266 | DV457721 | tri－ | CTCTGAGAATTGAACCATCC | GGGAACAAAGAAATTACTGG | 54 | 378 |
| JESR-093 | CESR-0282 | CK651527 | tri－ | CACCTCCCATTAGGGTTT | CTAATCGACGCTGATAATCC | 54 | 239 |
| JESR-094 | CESR-0297 | DV449646 | tri－ | AAACCACCTGCTAATGATGT | GCCTCTTCACTGCTAAAGAA | 54 | 129 |
| JESR-095 | CESR-0319 | CK649172 | tri－ | AATGAGTCTGACAATCAGGG | GCATGCTCTGTTCTGCTT | 54 | 336 |
| JESR-096 | CESR-0328 | CK651737 | tetra－ | ACACAAACACAATCAACAGC | CGCGACTCACTTTGTATGTA | 54 | 244 |
| JESR-097 | CESR-0335 | CK646328 | di－ | ACCGCTTCTTCTTTCTCTCT | TAGCCGGCAATATACAGAAT | 54 | 301 |
| JESR-098 | CESR-0349 | DV457928 | tri－ | AGATCACAAGGATCACAAGG | GCAGTTGTCAAACACTAGCA | 54 | 290 |
| JESR-099 | CESR-0365 | DV452331 | tri－ | ATAATGGCAAACAAGTGGTC | TGGTAGTGTTGTTCTTGCAG | 54 | 305 |
| JESR-100 | CESR-0373 | DR086161 | tri－ | ATCCACTTCTCTTCTTGTGC | ATTCTTGTCGAAGAGGTCAA | 54 | 180 |
| JESR-101 | CESR-0376 | DV451306 | tri－ | ATCCTAACACAGTTGCCATC | AAACTCAACCAAACCACAAC | 54 | 230 |
| JESR-102 | CESR-0378 | DV450115 | di－ | ATCCTTCTGCAGTAGCCATA | TTATATGCTACACATCAACCTG | 54 | 278 |
| JESR-103 | CESR-0397 | DN239725 | di－ | CAAGTTCGAGGAGTACAAGG | TGTTACAACGAGATGAGTGC | 54 | 292 |
| JESR-104 | CESR-0420 | DR083993 | tri－ | CCACAGTTCATCCTCAATTT | GATATTCACTCTGGAACCCA | 54 | 308 |
| JESR-105 | CESR-0423 | DV444684 | tetra－ | CCAGGTCCTTATCATTTCAA | CAAGCTAGGTCTGCAACTTT | 54 | 151 |
| JESR-106 | CESR-0426 | DV443850 | di－ | CCATTTGATTTGATGGAGTT | CAAGCCATTCTCCTACTCAC | 54 | 177 |
| JESR-107 | CESR-0432 | DV447786 | di－ | CCTGTGTAGAATCGTCCTTT | AACCAGAACCAATCTCAATG | 54 | 246 |
| JESR-108 | CESR-0441 | DV444811 | di－ | CTAGTAGAGCAGGTGTTGGG | CATCCCACTCAACAATTCA | 54 | 167 |
| JESR-109 | CESR-0604 | CK650239 | tri－ | AAAGAGGCTGGAGGAGGT | TCAACAGTGATCACAAGGAA | 54 | 219 |
| JESR-110 | CESR-0630 | CK647206 | tri－ | AAGGAGGTTATGGAGAGAGG | ATATTTCTTGCGGCTATGAC | 54 | 191 |
| JESR-111 | CESR-0631 | CK643600 | tri－ | AAGTGAGAGAGATGAGGCAA | CAATGCTAGAAATCCCAGAG | 54 | 113 |
| JESR-112 | CESR-0658 | DV451277 | tetra－ | ACTGCTACTCTAATGGGTGC | TTGGTTCATCCAAGAAAGTT | 54 | 179 |
| JESR-113 | CESR-0671 | DV448855 | di－ | AGAGGTTAGCAACATGGGTA | AGCATTTCAGTATGAAGACA | 54 | 225 |
| JESR-114 | CESR-0675 | DV444397 | tetra－ | AGATTGTGGATGGAGAAGTG | CGGAACCCACAAACATAATA | 54 | 310 |
| JESR-115 | CESR-0728 | DV457587 | penta－ | ATTGGCCTTCACAGAATATG | CATCAACACACGCTACACTT | 54 | 186 |
| JESR-116 | CESR-0795 | DV456336 | tetra－ | CCTTCTGACCGTAGTTTCTG | GTGCAGCTACTCCAAGAATC | 54 | 225 |
| JESR-117 | CESR-0802 | DV443286 | tri－ | CGGAAAGCTGAAGAAATAGA | TCTCAACGAAACATTCTCCT | 54 | 185 |
| JESR-118 | CESR-0806 | DV443221 | di－ | CTAAAGGCTGTGAAGAAGGA | TCCGAGCCAATTTCTTATTA | 54 | 276 |
| JESR-119 | CESR-0849 | DV458926 | tri－ | GAAGATGATTCTGGAGGACA | TCTCAAGAGATTCGTCGTCT | 54 | 286 |
| JESR-120 | CESR-0850 | CK645387 | tri－ | GAAGTTCTACAAGCATTGCC | CTACCACCACTCCTGTCACT | 54 | 113 |
| JESR-121 | CESR-0860 | DV456911 | tri－ | GAGAAGCTCTGTGAAGCTGT | TCATTTAATGGTTGGTTTGG | 54 | 204 |
| JESR-122 | CESR-0944 | DV444155 | tri－ | TAATGGCAATATCTCCTGCT | TGTCTTCAATCCACTTCTCC | 54 | 368 |
| JESR-123 | CESR-0993 | DV457789 | tri－ | TGAACTGTTGCCAGTACAAGA | TCAAGTTTAAGTGAAGGCAG | 54 | 359 |
| JESR-124 | CESR-1015 | DV448798 | tri－ | TTAAAGACGAATCAAGGGAG | ACCTTAACTCTTGGCACTCA | 54 | 202 |
| JESR-125 | CESR-0005 | DV441775 | di－ | GTTGAGAAGGAAACTGCTTG | ATAACAGCAAGACCATCACC | 55 | 284 |
| JESR-126 | CESR-0164 | CK643198 | tetra－ | TCCTCTCAATCCCTACCTCT | TGGATTCGGGTAGTGTTATC | 55 | 304 |
| JESR-127 | CESR-0157 | CK650031 | tri－ | AGCGAGGAGTTCTTATGTGA | GCAAACAAACAGGAGGTAAC | 55 | 367 |
| JESR-128 | CESR-0159 | DV444613 | di－ | TGATGACTGGGAAGGATTAG | TGGAGATTGATAGGGTATCG | 55 | 352 |
| JESR-129 | CESR-0288 | CK648120 | tri－ | AGATCCAGAACCCTTCACTT | TAACAAGAGGTCCAGCAACT | 55 | 206 |
| JESR-130 | CESR-0385 | CK643516 | di－ | ATTAAAGCCAACTTGGGTAA | GGATTAGAGCAGACATTTGG | 55 | 291 |
| JESR-131 | CESR-0425 | CK651507 | tri－ | CCATCTTCCCTCTACCTTCT | GCTTTGCTCTTGCTTTGTAT | 55 | 129 |
| JESR-132 | CESR-0430 | DR084342 | tri－ | CCTACCAATGAAAGTGGGTA | CTCGATATCCAGAATCTTGC | 55 | 387 |
| JESR-133 | CESR-0438 | DV447125 | di－ | CGGATCGAGTTCTATCTCAG | TCATGAGAGCTCTTTCCATT | 55 | 311 |
| JESR-134 | CESR-0439 | DN239293 | di－ | CGTGGCGGTACACACACA | CTCCCATTTGTTCTTCACAT | 55 | 311 |
| JESR-135 | CESR-0468 | DR084389 | di－ | GAGAACTGAAGATCGAATGC | CCACCCATAAGATGGTAATG | 55 | 245 |
| JESR-136 | CESR-0487 | DV457545 | tri－ | GCAACGAGAAATCTATGAGG | CCTCCATAACCACCTTCAC | 55 | 184 |
| JESR-137 | CESR-0499 | DV450349 | tri－ | GCTCTTACTCGTGTGGTTTC | AACCCTCTATAAACCCTTGG | 55 | 266 |
| JESR-138 | CESR-0529 | CK644884 | tri－ | GTCGCTGCTAGATCTTGACT | CGAAGATCGAAGAAGTGATT | 55 | 106 |
| JESR-139 | CESR-0530 | CK646783 | tri－ | GTCTCCCTCGCTCTAATTG | TGTAAACGATCAGACCACAA | 55 | 137 |
| JESR-140 | CESR-0576 | DV451121 | tri－ | TGTGGCAGTACTACAACGAG | ATGGCAGTCACAATTAAACC | 55 | 183 |
| JESR-141 | CESR-0584 | CK652079 | di－ | TTCTTTCTCTCTCTTTCTCCC | GATTAATCTGCTCGTCAAGG | 55 | 263 |
| JESR-142 | CESR-0685 | DV454511 | tetra－ | ACTGCTACTCTAATGGGTGC | TTGGTTCATCCAAGAAAGTT | 55 | 320 |
| JESR-143 | CESR-0713 | CK651958 | tri－ | ATCTCTGTCTCTGTCTGCGT | TTCATCACTCCCATTGAAAC | 55 | 353 |
| JESR-144 | CESR-0765 | DV447522 | tetra－ | CATTCCCGAGGATAAGTATG | CCTTCCTATCACTGAACCTG | 55 | 190 |
| JESR-145 | CESR-0772 | CK643741 | tri－ | CCACACTTGAAAGCTAAACC | TCTCTTGGCTGATCAGAGTT | 55 | 367 |
| JESR-146 | CESR-0794 | DV446367 | tri－ | CCTTAGGACGTCTCTCGTTT | AAGCTGCTTAGCAAATTGAC | 55 | 376 |
| JESR-147 | CESR-0827 | DV450516 | tri－ | CTGTAAGCGCTTCCTTTCTA | TATCAGGCCTATCCTCAAGA | 55 | 245 |
| JESR-148 | CESR-0885 | DV450974 | tri－ | GCGACAAGAAGAAGAAGAAG | GCAGTTGTCAAACACTAGCA | 55 | 180 |
| JESR-149 | CESR-0886 | CK645484 | tri－ | GCTATTCCTGTTGGTAATGC | TCTTCATCCTCATCATCCTC | 55 | 247 |
| JESR-150 | CESR-0888 | DV442383 | tri－ | GCTCAGTCTTTCAATTCACC | GAATTAACTCTCAGGCATGG | 55 | 214 |
| JESR-151 | CESR-0926 | CK648934 | di－ | GTCACTGTCTTCCTCCTCAA | AATACATTGACCTGATGGGA | 55 | 242 |
| JESR-152 | CESR-0935 | DV441362 | tri－ | GTTCACTGGATGTTAAGGGA | ACATTTCAGACATCGAGAGC | 55 | 329 |
| JESR-153 | CESR-0054 | DV440943 | tri－ | ATTAGACCTGCACTCAAGGA | GAAGGCTGTAGTTCCTGTTG | 56 | 122 |
| JESR-154 | CESR-0204 | DV456331 | tri－ | AGATGCCCAACCCTCCAC | TGCTCTAGTTCCTCCATGTT | 56 | 255 |
| JESR-155 | CESR-0233 | DV447790 | tetra－ | GATGAGGTGATACTGGCATT | CACTGCATTCAGCAGTAAGA | 56 | 300 |
| JESR-156 | CESR-0334 | DV458486 | di－ | ACATTGCTTCTCTGTTAGCC | AGGAGTTGAAGGACAGTGTG | 56 | 152 |
| JESR-157 | CESR-0336 | CK647260 | di－ | ACCGCTTCTTCTTTCTCTCT | TAGAAGGAAGACCCAACTCA | 56 | 281 |
| JESR-158 | CESR-0360 | DV442337 | tri－ | AGGATGTAGACGCTCTGGTA | CTCGGTGTAGAAGTTGAAGC | 56 | 232 |
| JESR-159 | CESR-0262 | DV447297 | di－ | CCCTCCTTCACTCTAACTCA | CAGGCTAAACTACCCATTTG | 56 | 390 |
| JESR-160 | CESR-0281 | DV452539 | tri－ | ACTCAGCATTGATGGATACC | AGCTCTCAACTCTTGCTCTG | 56 | 243 |
| JESR-161 | CESR-0314 | DV455407 | tri－ | AAGAAGTGTATGGGTTGCAC | TACGATACCTAGGGCTACGA | 56 | 323 |
| JESR-162 | CESR-0341 | DV442397 | tri－ | ACTGATGGGTATGTGAGAGG | TTCTTCATCATGGCTACCTT | 56 | 220 |
| JESR-163 | CESR-0406 | CK650685 | tri－ | CAGAAACGGAGAGGTCTG | AGATTGGAAGAGGAGAGGAG | 56 | 144 |
| JESR-164 | CESR-0350 | DV454821 | tri－ | AGCCCAGTCTCGCGGAAG | CAGTTCCCTTCAGAAGCTC | 56 | 231 |
| JESR-165 | CESR-0354 | DV456377 | tri－ | AGCGGAGGCGGAGGAAAT | AGCGGAGGCGGAGGAAAT | 56 | 297 |
| JESR-166 | CESR-0359 | DV445961 | tri－ | AGGATGTAGACGCTCTGGTA | GTAGGCATGGCTAGTGTTTC | 56 | 335 |
| JESR-167 | CESR-0431 | DV454676 | tri－ | CCTCCTGAGAGTAACCCTTT | TCTCCACCCTCATACACTTC | 56 | 235 |
| JESR-168 | CESR-0440 | CK643500 | tetra－ | CTAACGTACGGAGTCGTTTC | CCATCATCTTCTTCACCACT | 56 | 370 |
| JESR-169 | CESR-0454 | DV445263 | tetra－ | CTTCTGCAGCTACCCTACTG | GACAACTACAATCATAACACG | 56 | 394 |
| JESR-170 | CESR-0519 | DV441229 | tri－ | GGTAGTTGAATTGCTCAAGG | AACACCCACATGTACAGCTT | 56 | 237 |
| JESR-171 | CESR-0623 | DV442674 | penta－ | AAGATGTATGGTTGGTGAGG | GAGAAAGAGATGTGGGACAG | 56 | 262 |
| JESR-172 | CESR-0632 | CK648201 | tri－ | AAGTGGTACAGAGGCTCTCA | AGATCGGCTCAGATTCACTA | 56 | 327 |
| JESR-173 | CESR-0686 | CK641521 | di－ | AGGAAGAGGGAGAGGAAAG | TGTCATAACCTGCAGGGAC | 56 | 118 |
| JESR-174 | CESR-0754 | CK643845 | penta－ | CAGGCACTGGCCGAGAGA | GATCCATCTTATCGAGGTGA | 56 | 251 |
| JESR-175 | CESR-0770 | CK643469 | tri－ | CCAAGTAGGATCCTTGAGTG | GTCTCGACTGTATCCACCTC | 56 | 349 |
| JESR-176 | CESR-0821 | DR084352 | di－ | CTCTCACTCTCCTCCGTTTA | TGATTAATCCTGGGAGACAC | 56 | 358 |
| JESR-177 | CESR-0831 | DV456879 | di－ | CTTACACACCACCTTCAAGC | AGCACGGTAGAAAGACCATA | 56 | 219 |
| JESR-178 | CESR-0838 | DV458597 | tri－ | CTTTAGTCCACCTCAAGTGC | TGCAGCAATCAACTCTACTG | 56 | 375 |
| JESR-179 | CESR-0839 | CK643726 | tri－ | CTTTAGTCCACCTCAAGTGC | CATCCTCTTCTTCATCCTCA | 56 | 251 |
| JESR-180 | CESR-0871 | DV450004 | tri－ | GATTACACGACCTTTGTGGT | AGGGTAAGGAACAGTAAGGC | 56 | 383 |
| JESR-181 | CESR-0896 | DR087953 | tri－ | GCTTCTCTTGCTGATATGGA | CTCTGTTGTTACCCAGTGGT | 56 | 223 |
| JESR-182 | CESR-0947 | CK648462 | tri－ | TACACATGGAGGGCTACTCT | TCAGGAGTACCCTTCTGAAA | 56 | 179 |
| JESR-183 | CESR-0999 | CK651493 | tri－ | TGCAGCTAGAGTAGGGAAAG | GATTTCTGATCTTGGCTCTG | 56 | 258 |
| JESR-184 | CESR-1012 | CK645327 | tri－ | TGTTCTAGCTGTTGCAGTTG | ATGTCGTCATCAGACTCCTC | 56 | 192 |
| JESR-185 | CESR-0379 | DN238673 | tri－ | ATCTTGTAGACTCGGTGGTG | GGTATGCATTCAGTCCATTT | 57 | 152 |
| JESR-186 | CESR-0410 | CK643536 | tetra－ | CAGCTCAGTACTCTCTCTCTCTC | GCTTATCAGAATCAACAATCC | 57 | 195 |
| JESR-187 | CESR-0542 | DN239370 | di－ | TACTACATCCTTGCCACCTC | TAAACACACCGTCATCACAT | 57 | 157 |
| JSSR-188 | Ns.10 |  | - | GTCATTACGGCGAATCTGCT | CGATTTTGCGAATTACCACC | 55 | 202 |
| JSSR-189 | Ns.22 |  | - | CAGCCAAATCAACATCCCTT | CAAGCCCCATCATCATTTTC | 55 | 298 |
| JSSR-190 | Ns.235 |  | - | CCAAAACATAGGGAGCGAAA | AATTATGGGCAGGAGAAGCC | 55 | 210 |
| JSSR-191 | Ns.260 |  | - | TCAGCTGTATGTTGAGTGAGCA | AGGGAAGGAACACCTCTCCTA | 50 | 224 |
| JSSR-192 | Ns.308 |  | - | GGAAATTGGTTATGTCCTTTCC | CGCATTGGACTTCCTACAAA | 55 | 151 |
| JSSR-193 | Ns.391 |  | - | TCTCAACCTCAGAATGTTCCAA | ATTTCCTGCACCCGGATAA | 55 | 132 |
| JSSR-194 | Ns.622 |  | - | TAAGTCGCGCAAATCCTTCT | AGCCCACAACAACTGTGTGA | 55 | 147 |
| JSSR-195 | Ns.658 |  | - | CATGATGGCCCGAAGATAGT | TCGTTGGAGCCATTACATTTC | 55 | 234 |
| JSSR-196 | Ns.689 |  | - | AGGATGATGATGAGACAAGAAGA | CAGACTGGACTTGAACTTTCACT | 55 | 144 |
| JSSR-197 | Ns.717 |  | - | GCCAAATCGCCAAGGTAATA | GGTGAGTGATAAGGTTACGGC | 55 | 201 |
| JSSR-198 | Ns.720 |  | - | CCATTACTTACACATTGGACTTCCT | GGAAATTGGTTATGTCCTTTCC | 55 | 157 |
| JSSR-199 | Ns.774 |  | - | AACCCGCAGAGAATCATGG | TCTCTTGCTTCTGTCACAACG | 55 | 124 |
| JSSR-200 | Ns.978 |  | - | TGTTGGCCATATTTCCCATT | TTGAACACACTTGGCCAGAA | 55 | 238 |
| JSSR-201 | SSRY7 |  | di－ | TGCCTAAGGAAAATTCATTCAT | TGCTAAGCTGGTCATGCACT | 45 | 250 |
| JSSR-202 | SSRY50 |  | di－ | CCGCTTAACTCCTTGCTGTC | CAAGTGGATGAGCTACGCAA | 55 | 271 |
| JSSR-203 | SSRY100 |  | di－ | ATCCTTGCCTGACATTTTGC | TTCGCAGAGTCCAATTGTTG | 55 | 210 |
| JSSR-204 | SSRY107 |  | di－ | CCATTTTCTCTTGCTTCTGTCA | TGGTTTGAAGTCCTATAAAATCCTT | 45 | 120 |
| JSSR-205 | SSRY113 |  | di－ | TTTGCTGACCTGCCACAATA | TCAACAATTGGACTAAGCAGC | 45 | 187 |
| JSSR-206 | SSRY127 |  | di－ | CTTCGGCCTCTACAAAAGGA | GCTGAACTGCTTTGCCAACT | 45 | 130 |
| JSSR-207 | SSRY133 |  | di－ | AGCATGTCATTGCACCAAAC | CGACTGCATCAGAACAATGC | 45 | 295 |
| JSSR-208 | SSRY146 |  | - | TTCCCTCGCTAGAACTTGTC | CTATTTGACCGTCTTCGCCG | 45 | 139 |
| JSSR-209 | SSRY150 |  | - | CAATGCAGGTGAAGTGAATACC | AGGGTGCTCTTCAGAGAAAGG | 45 | 175 |
| JSSR-210 | SSRY151 |  | - | AGTGGAAATAAGCCATGTGATG | CCCATAATTGATGCCAGGTT | 45 | 182 |
| JSSR-211 | SSRY153 |  | - | TTCCAGAAAGACTTCCGTTCA | CTCAACTACTGCACTGCACTC | 45 | 117 |
| JSSR-212 | SSRY159 |  | di－ | CTTATCCTGTCCCCTCCACC | GACAATTGCATAGGAAGCACA | 45 | 159 |
| JSSR-213 | SSRY177 |  | di－ | ACCACAAACATAGGCACGAG | CACCCAATTCACCAATTACCA | 45 | 268 |
| JSSR-214 | SSRY185 |  | di－ | GAAGAAGACGGTTAAAGCAAGTT | ATGCCAGTTTGCTATCCAGG | 55 | 243 |
| JSSR-215 | SSRY4 |  | di－ | ATAGAGCAGAAGTGCAGGCG | CTAACGCACACGACTACGGA | 55 | 287 |
| JSSR-216 | SSRY46 |  | di－ | TCAGGAACAATACTCCATCGAA | CGCTAAAGAAGCTGTCGAGC | 55 | 268 |
| JSSR-217 | SSRY53 |  | di－ | CCATGCAGTAGTGCCATCTTT | ATTTTCACCAACCGCAACTC | 55 | 138 |
| JSSR-218 | SSRY57 |  | di－ | TGTCATTGTCTGTTGACCATTT | TAACCTGCCAAGAACAAGGC | 55 | 293 |
| JSSR-219 | SSRY58 |  | di－ | GAAGGACAAGCAAAGAAGCAA | TGGAATCCAATATTGATGACTAAGA | 55 | 217 |
| JSSR-220 | SSRY61 |  | di－ | GGCTGCTTTACCTTCTACTCAGA | CAAGAACGCCAATATGCTGA | 55 | 233 |
| JSSR-221 | SSRY63 |  | di－ | TCAGAATCATCTACCTTGGCA | AAGACAATCATTTTGTGCTCCA | 55 | 290 |
| JSSR-222 | SSRY65 |  | di－ | CATCGCCAAATCGTCAAGTA | TGATGCCATGCATTTCACTT | 55 | 299 |
| JSSR-223 | SSRY80 |  | di－ | TTCCTGGAAATGTCCTTAGATG | TGGCACATGCAACAATTAGC | 55 | 299 |
| JSSR-224 | SSRY109 |  | di－ | TGCTAATTGCAGGAAATAGGAT | GCAGCTTTTTAGCATAACAATCAA | 55 | 125 |
| JSSR-225 | SSRY112 |  | di－ | CGCAAGGTAAATCGGAGCTA | ACAATCAAAGGAGTCGTGTAATC | 55 | 117 |
| JSSR-226 | SSRY114 |  | di－ | AACAGGAAGGAAAATCAAGCC | TCAACTGCAGATTCATTCAAGA | 55 | 167 |
| JSSR-227 | SSRY117 |  | di－ | TAAAGTTTGGCATGCCTGTG | GCAAATGTGTTTTCAATATAAGGC | 55 | 142 |
| JSSR-228 | SSRY118 |  | di－ | TAGAGCAGCTGCAAAGCAAA | TCGTTTTCCTGTTGAAATCTTG | 55 | 169 |
| JSSR-229 | SSRY119 |  | di－ | AACATAGGCATTAAAGTTTGGCA | GCAAATGTGTTTTCAATATAAGGC | 55 | 155 |
| JSSR-230 | SSRY123 |  | di－ | AGCAGATCCAAATCACTGAAA | TTCAACAATAAAGCTCAGAAAGAG | 55 | 136 |
| JSSR-231 | SSRY124 |  | di－ | CTGCTGGACGGAGGATTCTA | TGGCATCAATTTTTGCTTCA | 55 | 146 |
| JSSR-232 | SSRY125 |  | di－ | CAGGACATGACGCAATTCTG | GCATGTTAGAAGTTTTTGCAATTT | 55 | 247 |
| JSSR-233 | SSRY129 |  | di－ | CTTTTTGCCAGTCTTCCTGC | AATGGATCATGTTCAATGTCTTC | 55 | 205 |
| JSSR-234 | SSRY132 |  | di－ | CTTTTTGCCAGTCTTCCTGC | TGTCCAATGTCTTCCTTTCCTT | 55 | 196 |
| JSSR-235 | SSRY134 |  | di－ | TCCACAAAGATAAGCTAAGCG | GCAAGTTCAAAAGGAGCAGC | 55 | 213 |
| JSSR-236 | SSRY136 |  | di－ | CGACTGCATCAGAACAATGC | AGCATGTCATTGCACCAAAC | 55 | 296 |
| JSSR-237 | SSRY140 |  | tri－ | CAGTGAGCAGAAACTAAAAACATTG | GGCACTTTGGAAAGGAAGAG | 55 | 212 |
| JSSR-238 | SSRY141 |  | di－ | TCCAAAATCTTGGTCATTTTGA | TGCTGTGATTAAGGAACCAACTT | 55 | 262 |
| JSSR-239 | SSRY155 |  | - | CGTTGATAAAGTGGAAAGAGCA | ACTCCACTCCCGATGCTCGC | 55 | 158 |
| JSSR-240 | SSRY158 |  | di－ | CCTTACTTGTGTTTCTTACTGACAAG | CCAAGTCCTCACCTCCAAAG | 55 | 224 |
| JSSR-241 | SSRY184 |  | tri－ | TCATCCCAAAAATACCTCTAACA | CTCCGACAAGCATGTGAATG | 55 | 163 |
